# 

**Published:** 1917-07

**Authors:** 


Yearly Vol. 72 July 1917 Number 12

### Buffalo Medical Journal

Established 1845 by AUSTIN FLINT, M.D.

EDITOR AND PUBLISHER:

A. L. BENEDICT, A.M., M.D., 228 Summer Street, Buffalo, N. Y.

LIBRARY
JUL 14 1917
SURGEON GENERAL'S OFFICE

ASSOCIATE EDITORS:

New York State:

Grover W. Wende, M.D., Buffalo.

C. W. Hennington, B.S., M.D., Rochester.

Charles Haase, M.D., Elmira.

Willis E. Ford, M.D., Utica.

Martin B. Tinker, B.S., M.D., Ithaca.

H. I. Davenport, A.M., M.D., Auburn

J. J. Buettner, M.D., Syracuse.

Foreign:

Sir William Osler, M.D., LL.D., Oxford, Eng.

Prof. P. K. Pel, M.D., Amsterdam, Holland.

Rene Gaultier, M.D., Paris, France.

J. George Adami, M.D., LL.D., F.R.S., Montreal, Can.

Henry W. Hill, LL.D., Buffalo, Associate Editor in Medical Jurisprudence.

